# Role of the Intravoxel Incoherent Motion Diffusion Weighted Imaging in the Pre-treatment Prediction and Early Response Monitoring to Neoadjuvant Chemotherapy in Locally Advanced Breast Cancer

**DOI:** 10.1097/MD.0000000000002420

**Published:** 2016-01-29

**Authors:** Shunan Che, Xinming Zhao, Yanghan OU, Jing Li, Meng Wang, Bing Wu, Chunwu Zhou

**Affiliations:** From the Department of Diagnostic Radiology, Cancer Hospital, Chinese Academy of Medical Sciences, Peking Union Medical College(SN C, XM Z, YH O, J L, CW Z); Department of Epidemiology, Cancer Hospital, Chinese Academy of Medical Sciences, Peking Union Medical College(M W); and GE MR Research China(B W), Beijing, PR China.

## Abstract

The aim of this study was to explore whether intravoxel incoherent motion (IVIM) diffusion-weighted imaging (DWI) can probe pre-treatment differences or monitor early response in patients with locally advanced breast cancer receiving neoadjuvant chemotherapy (NAC).

Thirty-six patients with locally advanced breast cancer were imaged using multiple-b DWI with 12 b values ranging from 0 to 1000 s/mm^2^ at the baseline, and 28 patients were repeatedly scanned after the second cycle of NAC. Subjects were divided into pathologic complete response (pCR) and nonpathologic complete response (non-pCR) groups according to the surgical pathologic specimen. Parameters (D, D^∗^, f, maximum diameter [MD] and volume [V]) before and after 2 cycles of NAC and their corresponding change (Δparameter) between pCR and non-pCR groups were compared using the Student *t* test or nonparametric test. The diagnostic performance of different parameters was judged by the receiver-operating characteristic curve analysis.

Before NAC, the f value of pCR group was significantly higher than that of non-pCR (32.40% vs 24.40%, *P* = 0.048). At the end of the second cycle of NAC, the D value was significantly higher and the f value was significantly lower in pCR than that in non-pCR (*P* = 0.001; *P* = 0.015, respectively), whereas the D^∗^ value and V of the pCR group was slightly lower than that of the non-pCR group (*P* = 0.507; *P* = 0.676, respectively). ΔD was higher in pCR (−0.45 × 10^–3^ mm^2^/s) than that in non-pCR (−0.07 × 10^−3^ mm^2^/s) after 2 cycles of NAC (*P* < 0.001). Δf value in the pCR group was significantly higher than that in the non-pCR group (17.30% vs 5.30%, *P* = 0.001). There was no significant difference in ΔD^∗^ between the pCR and non-pCR group (*P* = 0.456). The prediction performance of ΔD value was the highest (AUC [area under the curve] = 0.924, 95% CI [95% confidence interval] = 0.759–0.990). When the optimal cut-off was set at −0.163 × 10^−3^ mm^2^/s, the values for sensitivity, specificity, positive predictive value (PPV), and negative predictive value (NPV) were up to 100% (95% CI = 66.4–100), 73.7% (95% CI = 48.8–90.9), 64.3% (95% CI = 35.6–86.0), and 100% (95% CI = 73.2–99.3), respectively.

IVIM-derived parameters, especially the D and f value, showed potential value in the pre-treatment prediction and early response monitoring to NAC in locally advanced breast cancer. ΔD value had the best prediction performance for pathologic response after NAC.

## INTRODUCTION

Neoadjuvant chemotherapy (NAC) is the standard treatment option for patients with locally advanced breast cancer^1^. The major clinical benefit for NAC compared with adjuvant therapy is the downstaging of large tumors and the increased rates of breast-conserving surgery.^2^ The response to breast cancer chemotherapy has been shown to correlate with long-term outcomes, although only a pathologic complete response (pCR) has a major impact on disease-free survival and overall survival.^3^ However, only a minority of patients (about only 13%) were featured with pCR due to the heterogeneity of breast cancer.^4,5^ It is crucial to assess the response of tumors before the treatment or at an early therapy stage, which could facilitate treatment planning for individual patient; it may also help to avoid side effects and improve the cost-effectiveness of NAC.

Magnetic resonance imaging (MRI) has been shown to be superior to physical examination, mammography, or ultrasonography in therapeutic response evaluation of breast cancer to NAC.^6,7^ Currently, the assessment of the size and volume of residual tumor based on contrast-enhanced MRI (DCE-MRI) is the most commonly used indicator for monitoring tumor response after NAC.^8,9^ However, DCE-MRI often overestimates the extent of lesion due to surrounding scar, necrosis, fibrosis, and reactive inflammation caused by tumor response.^10,11^ Partial volume effects of very small foci of residual tumor and the antivascular effect of certain chemotherapy drugs may lead to underestimation of tumor size using DCE-MRI.

Diffusion-weighted imaging (DWI) has been considered as a potential method to overcome the limitations of traditional DCE-MRI assessment that depends on an inherent special tissue contrast mechanism.^12^ DWI probes the microscopic motion of water molecules under the influence of applied diffusion gradient. The diffusion of the water molecules is sensitive to the change of tissue microstructure, including tissue cellularity and the integrity of cell membranes.^13^ Apparent diffusion coefficient (ADC), which is calculated using the traditional monoexponential model, can be used to quantitatively reflect the diffusion of tissue water molecules. Nevertheless, the pure molecular diffusion and microcirculation perfusion simultaneously contributed to the ADC value, and this may hurdle its ability in characterizing the tissue microstructure. The intravoxel incoherent motion (IVIM) theory, first described by Le Bihan et al,^12^ makes it possible to separate the pure water diffusion and microcirculation perfusion of the tissue using the multiple-b value DWI data. Perfusion-related diffusion (D^∗^) and perfusion fraction (f), related to perfusion of blood flow in tissue, can be obtained without application of contrast medium.

This IVIM approach has been shown to be useful for the differentiation of various benign and malignant tumors.^14–17^ Several studies have proved the potential value of IVIM model in monitoring the chemotherapy response for various types of malignant tumors, such as hepatocellular carcinoma,^18^ head and neck carcinomas,^19^ nasopharyngeal carcinoma,^20^ and so on. To our knowledge, no previous attempt has been made to assess the value of DWI IVIM model in the evaluating the therapeutic effect of breast cancer. In this study, the value of IVIM-derived parameters in pretreatment prediction and early response monitoring to NAC in locally advanced breast cancer are investigated.

## MATERIALS AND METHODS

### Ethical Statement

The study was approved by the Regional Ethical Committee at Cancer Hospital, Chinese Academy of Medical Sciences, Peking Union Medical College. The informed consent was obtained from each patient before MRI examination.

### Patients

From March 2014 to May 2015, eligible subjects with breast cancer were screened from 58 patients receiving NAC and breast MRI examinations concurrently. Patients enrolled for this study met the following criteria. First, all patients had core needle biopsy confirmed histologically invasive breast cancer at least 2 cm in the longest dimension or of any size with exact evidence of axillary lymph node metastases. Second, the MRI scans should be performed before NAC and/or after 2 cycles (within 1–3 days before 3 cycles) of the chemotherapy. Third, subjects had accepted a completed NAC and subsequently received either breast conserving surgery with axillary nodal clearance or modified radical mastectomy. Patients were excluded if they had following situations: difficulties in evaluating DWI images due to obvious artifacts; pregnancy or any MRI contraindications (including metal implants, cardiac pacemaker, heart metal stent, and so on); and allergic reaction to MRI contrast medium. The NAC regimens consisted of paclitaxel with epirubicin, or paclitaxel with carboplatin once every 3 weeks. All patients were treated with 4 to 8 cycles according to their regimen protocol and physical situation.

### MR Imaging

MRI was performed using a whole-body 3.0T MR scanner (Discovery MR750; GE Healthcare, MI). All patients were imaged in the prone position with an 8-channel phased-array breast coil. Patients fasted for at least 2 hours before imaging and avoided any strenuous exercise before the examination.

Conventional MR breast imaging included transverse T2-weighted imaging fat suppression, transverse DWI fat suppression with short time inversion recovery (STIR) (b = 0,1000 s/mm^2^), transverse 3D Vibrant-Flex multi-phase dynamic contrast-enhancement (DCE-MRI), and sagittal 3D Vibrant-Flex for delayed acquisition. For the DCE-MRI sequence, transverse 3D Vibrant-Flex was scanned before and repeated 9 times (duration 45 s each) after intravenous administration of 0.1 mmol/kg Gd-DTPA (Magnevist; Bayer, Berlin, Germany) at 2 mL/s (followed by a flush of 20-mL saline solution) via a power injector with a 15-second timing delay.

Transverse multiple-b DWI was acquired using an STIR fat-saturated, single-shot, spin-echo echo-planar imaging (SE-EPI) before the DCE-MRI. Twelve b values were used: 0, 10, 20, 30, 50, 70, 100, 150, 200, 400, 800, and 1000 s/mm^2^. Number of excitation (NEX) was 1, 3, 3, 3, 3, 2, 2, 2, 2, 3, 5, and 6, respectively. The corresponding parameters were as follows: the repetition time/echo time, 2400/62.1 ms; field of view, 320 × 320 mm; matrix size, 128 × 160; section thickness, 5 mm; intersection gap, 1 mm; flip angle, 90°; receiver bandwidth, 250 kHz; and parallel imaging (ASSET) factor, 2. The imaging duration of the multiple-b DWI sequence was 8 minutes 19 seconds.

### MRI Data Acquisition and Image analysis

On account of a biexponential algorithm model of IVIM theory, which was described by Le Bihan et al,^12^ the relationship between signal intensity (S) and b-factor was expressed by the following equation (Eq. 1): 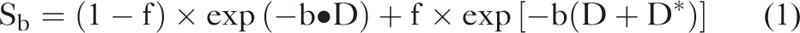


in which D is the true diffusion coefficient that reflected the pure molecular diffusion, f is the perfusion fraction representing the volume fraction of microcirculation, and D^∗^ is the pseudo-diffusion coefficient related to perfusion-related diffusion, S_b_ is the signal intensity in the pixel with diffusion gradient b, and S_0_ is the signal intensity at b = 0.

Image analysis was performed automatically by the workstation (Advantage Workstation 4.6; GE Healthcare) with software (Function tools MADC, GE Healthcare) and fitted on a pixel-by-pixel basis using the Levenberg–Marquardt algorithm.^21^ D, D^∗^, and f were calculated consecutively, where D was obtained by a simplified linear fit equation (Eq. 2) using b values >200 s/mm^2^:. 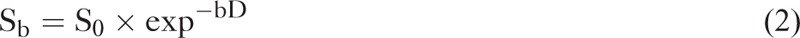


This was based on the assumption that D^∗^ is obviously larger than D. When the b-factor is bigger than 200 s/mm^2^, the effects of D^∗^ on the signal can be neglected.

Ultimately, the corresponding f and D^∗^ can be calculated primitively from Eq. (1) by using a nonlinear regression algorithm.

**FIGURE 1:**
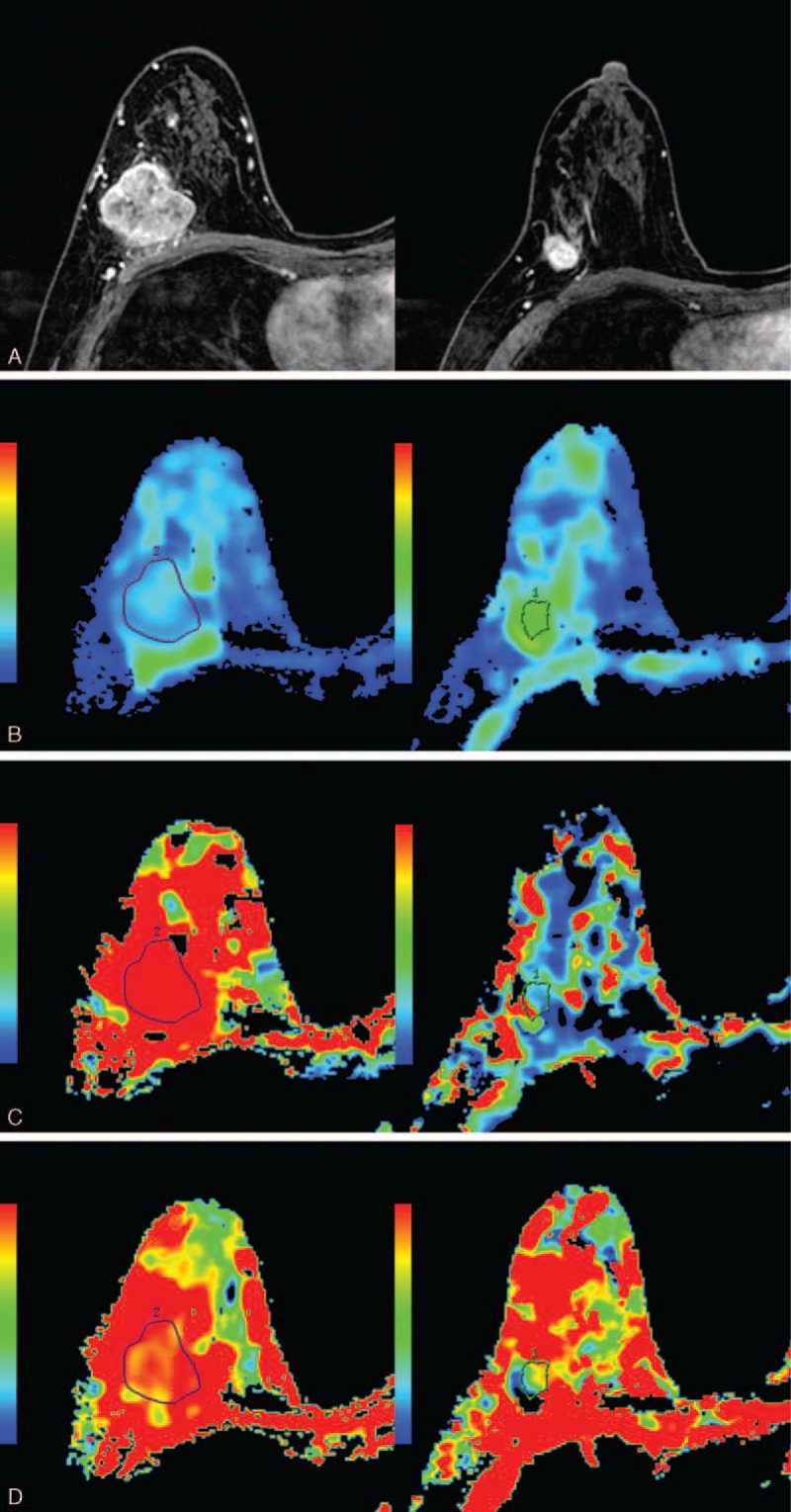
A 51-year-old woman with invasive ductal carcinoma who was enrolled into the pCR group. Images in each vertical row are from 2 measurement time-points: before NAC (NAC-pre) and after 2 cycles of NAC (NAC-mid). A, The tumor shrunk obviously after 2 NAC cycles. B, The D value increased significantly from 0.906 × 10^–3^ to 1.310 × 10^–3^ mm^2^/s after the therapy. C, The D^∗^ value decreased significantly from 40.4 × 10^–3^ to 13.8 × 10^–3^ mm^2^/s after 2 cycles of NAC. D, The f value significantly decreased to 14.2% after the NAC initiation, which was 35.7% before NAC.

**FIGURE 2:**
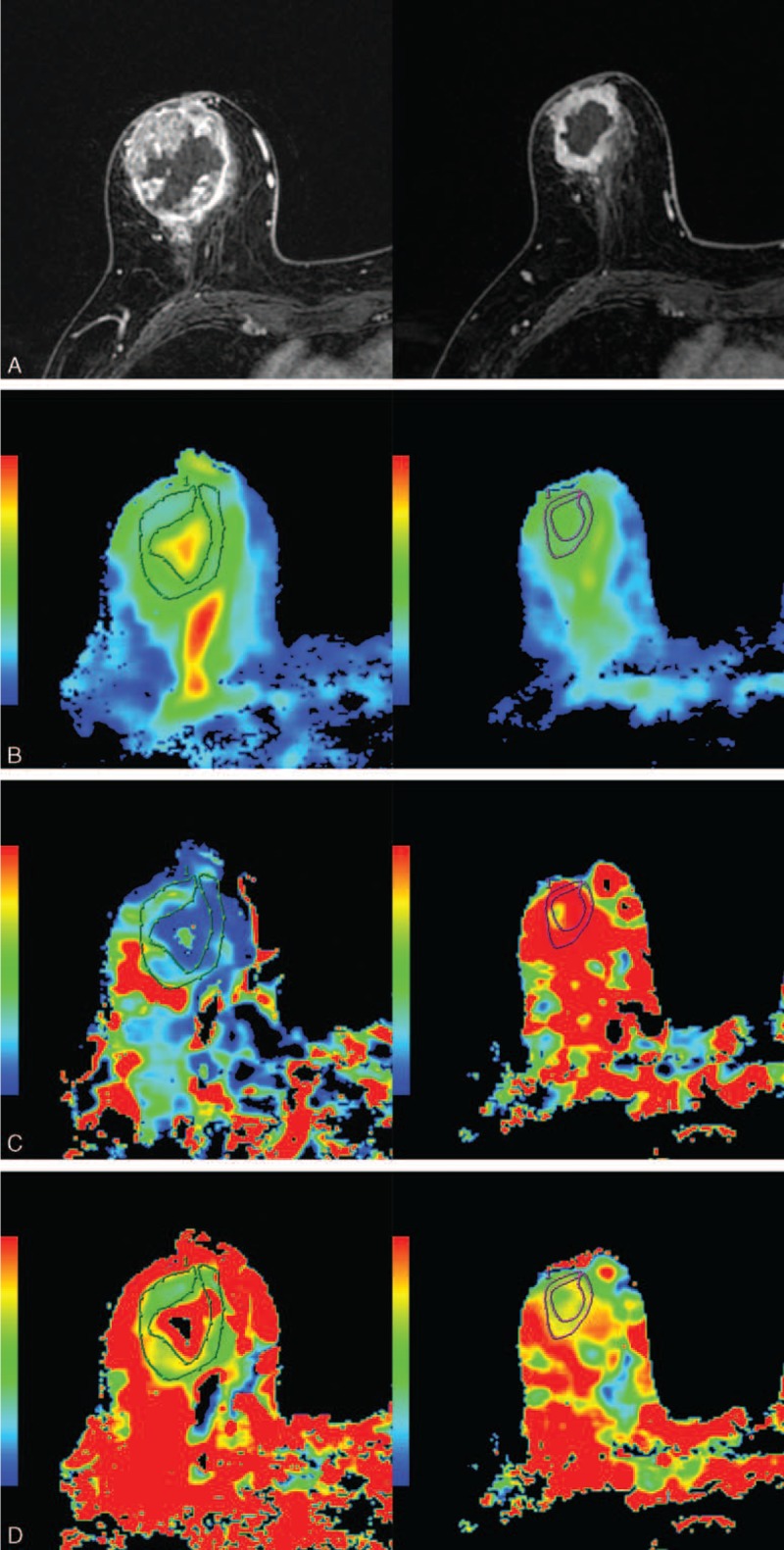
A 75-year-old woman with invasive ductal carcinoma who was classified as the non-pCR group. Images in each vertical row are from 2 measurement time-points: before NAC (NAC-pre) and after 2 cycles of NAC (NAC-mid). A, The tumor reduced mildly after 2 NAC cycles. B, The D value increased slightly from 1.110 × 10^–3^ to 1.410 × 10^–3^ mm^2^/s after the therapy. C, The D^∗^ value increased from 14.8 × 10^–3^ to 27.4 × 10^–3^ mm^2^/s after 2 cycles of NAC. D, The f value slightly decreased to 21.4% after the NAC initiation, which was 29.2% before NAC.

The region of interest (ROI) analysis on the parametric maps was performed by 2 radiologists who had 5 and 12 years of MRI diagnosis experience, respectively. T2WI and DCE-MR images were used as references to determine the extents of lesion on the corresponding IVIM parametric maps. ROIs were manually drawn on the DWI image with b = 800 s/mm^2^. The level of maximum transverse diameter of the breast lesion was located, encompassing as much of the tumor area as possible, and avoiding the obvious necrotic, hemorrhagic, and cystic areas (Figures 1 and 2). In addition, the radiologists manually contoured the edge of the target lesions slice by slice on the DCE-MR images using the segment tool in the postprocessing workstation (Advantage Workstation 4.6; GE Healthcare); then, the volume was automatically calculated. Meanwhile, the transverse maximum diameters (MDs) were recorded as well. All parameters of each ROI were recorded and an average result was obtained by triplicated measurements.

### Pathologic Assessment of Response

An experienced pathologist blindly assessed all specimen slices. The histological response to chemotherapy was estimated using the Miller and Payne grading system^22^: grade 1, some change to individual malignant cells but no reduction in overall cellularity; grade 2, a minor loss of invasive tumor cells but overall cellularity is still high; grade 3, a considerable reduction in tumor cells, between an estimated 30% and 90% tumor cell loss; grade 4, a marked disappearance of invasive tumor cells such that only small clusters or widely dispersed cells could be detected, more than 90% reduction in tumor cell; and grade 5, no invasive tumor cells identifiable in the sections from the site of the previous tumor, only a little ductal carcinoma in situ, or tumor stroma remained. Grade 5 was supposed to represent a pathological complete response (pCR). All subjects were divided into a pCR group and a non-pCR group. The Grades 1 to 4 were classified into the non-pCR group, while Grades 5 were the pCR group correspondingly.

### Statistical Analysis

Kolmogorov–Smirnov test was used to estimate the distribution of the all parameters: normally distributed data are presented as mean ± standard deviation, while non-normal data are presented as median and interquartile range. Differences of the parameters (D, D^∗^, f, MD, and V) before NAC and after 2 cycles of NAC between the non-pCR and the pCR groups were compared using the Student *t* test or nonparametric test. Diagnostic performance for the different parameters and their corresponding changes in predicting the NAC treatment outcomes were assessed using the receiver-operating characteristic (ROC) curve analyses. Diagnostic accuracy was determined by calculating the AUC (0.5, no diagnostic accuracy; AUC = 0.5–0.7, poor diagnostic accuracy; AUC = 0.7–0.9, reasonable diagnostic accuracy; AUC > 0.9, very good diagnostic accuracy).^23^ The optimal cutoff values, prediction sensitivity, specificity, positive predictive value (PPV), and negative predictive value (NPV) were calculated according to the Youden index.

In order to reveal the relationship between the parametric measures and effects of NAC, the correlation between the quantitative parameters and the shrinkage of mass (%) after NAC were assessed using Spearman correlation test. All analyses were carried out using commercial software (PASW, version.19.0; SPSS, Chicago, IL; MedCalc Software, version 11.4.2.0, Mariakerke, Belgium). The methods of Hanley & McNeil were used for the calculation of the difference between different AUCs. All significance tests were 2-sided and a *P* value of less than 0.05 was considered statistically significant.

## RESULTS

### Clinical Characteristic of Enrolled Patients

Characteristics of enrolled patients are listed in Table 1. Out of the 58 patients who had received NAC and breast MR scans, 36 were included in this study, and the excluded 22 patients were due to lack of enough IVIM-DWI images (n = 6), image distortions (n = 5), surgery treatment before completed NAC (n = 4), distant metastasis (n = 3), giving up therapy after 1cycle of NAC (n = 2), and lack of proper pathologic results (n = 2). The patients ranged from 27 to 75 years old with a mean age of 50.9 years. Thirty-six persons completed the pretreatment MRI scan, while 28 completed the pretreatment and mid-treatment MRI examinations synchronously. The type of tumor histology before NAC and histological response to chemotherapy for all subjects are summarized in Table 1.

**TABLE 1:**
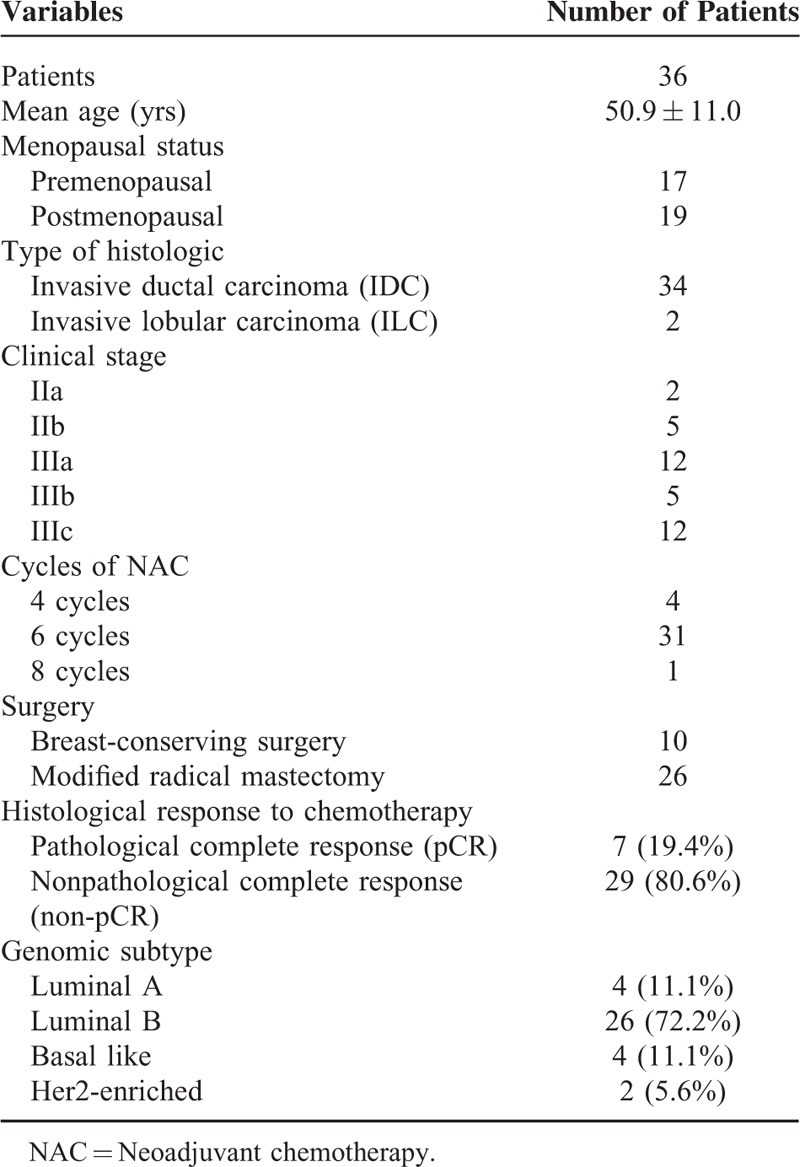
Characteristics of Enrolled Patients

### Parameters Before NAC and After 2 Cycles of NAC in Different Groups

The baseline D value was significantly lower than the D value after 2 cycles (*P* < 0.001), whereas baseline f value was significantly higher than the f value after 2 cycles of NAC (*P* < 0.001). No significant difference was observed on D^∗^ value between baseline and time after 2 cycles (*P* = 0.569) (Table 2).

**TABLE 2:**
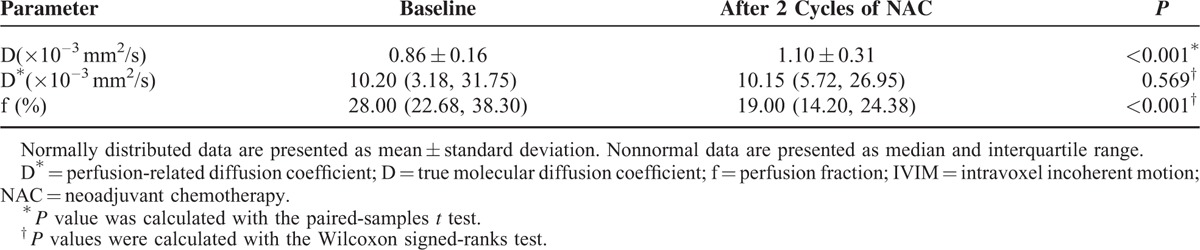
Parameters of IVIM Model During Baseline and After 2 Cycles of NAC

**FIGURE 3:**
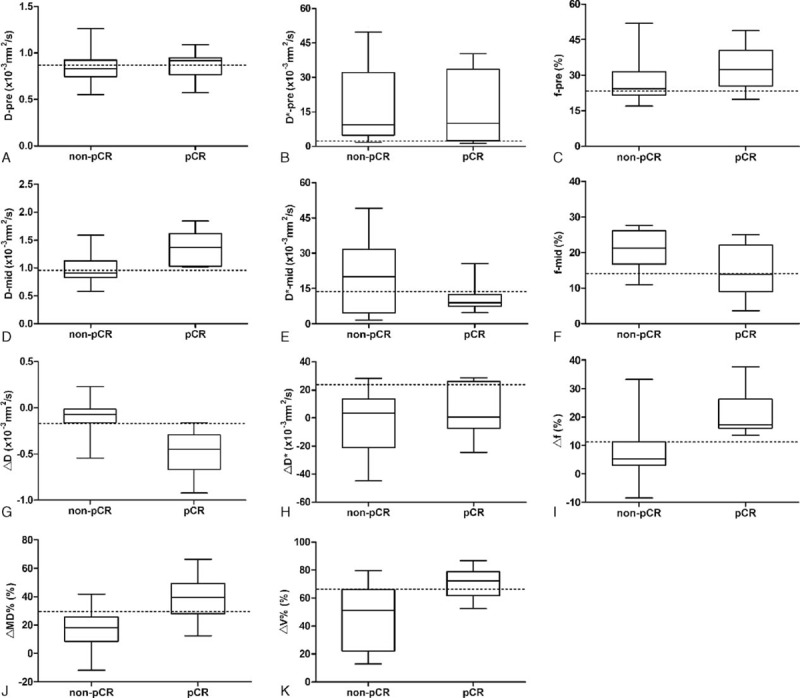
Box and whisker plot shows the parameters (D, D^∗^, and f) before and after 2 cycles of NAC, and their corresponding change (Δparameter) and the shrinkage of mass (ΔMD% and ΔV%) between pCR and non-pCR groups. The dotted line represents a cutoff value calculated by the ROC analysis. The f-pre and D-mid in pCR were significantly higher than that in non-pCR. The f-mid in pCR was significantly lower than that in non-pCR. The f, MD, and V values were decreased more in pCR than in non-pCR. By contrast, the D was increased more in pCR than in non-pCR.

The parameters (including D, D^∗^, f, MD, and V) before NAC and after 2 cycles of NAC between pCR and non-pCR are summarized in Table 3 and Figure 3. The f value of pCR group in pretreatment was significantly higher than that of non-pCR (*P* = 0.048). Meanwhile, no significant differences were found in D, D^∗^, MD, and V before NAC between the pCR and non-pCR group (*P* > 0.05).

**TABLE 3:**
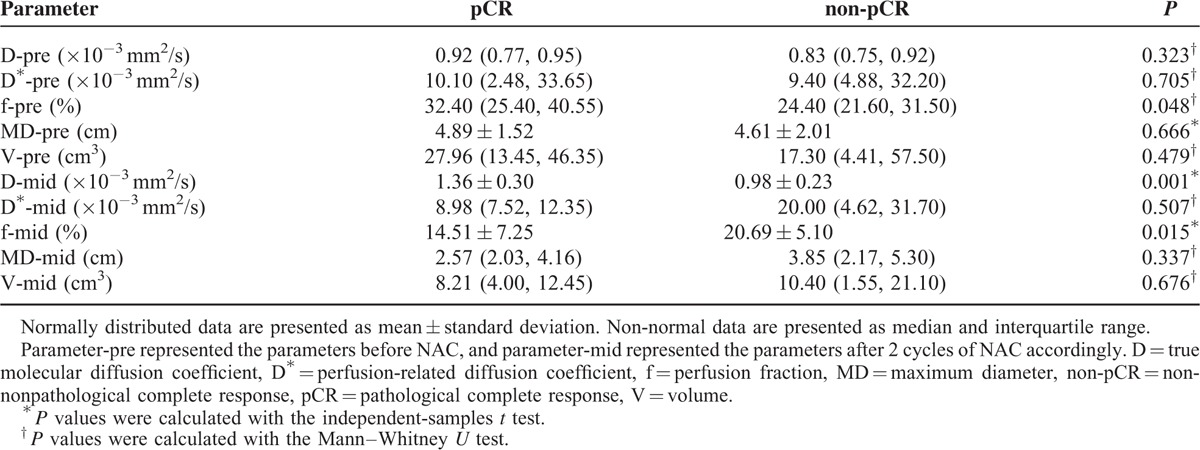
Parameters Before NAC and After 2 Cycles of NAC Between pCR and non-pCR

After 2 cycles of NAC, the D value of the pCR group was significantly higher than that of the non-pCR group (*P* = 0.001). The f value of the pCR group was significantly lower than that of the non-pCR group (*P* = 0.015). The D^∗^ of the pCR group was slightly lower than that of the non-pCR group, but there was some overlap between these 2 groups (*P* = 0.507). No statistically significant difference between the pCR and non-pCR groups in terms of the MD and V was observed (*P* = 0.337; *P* = 0.676, respectively).

### Change of the Parameters Between pCR and non-pCR

The growth of D was higher in pCR than in non-pCR in the mid-treatment (*P* < 0.001). The decrease of the f value in the pCR group was significantly higher than that in the non-pCR group (*P* = 0.001). There was no significant difference in the change of D^∗^ between the pCR and non-pCR (*P* = 0.456). A statistically significant difference in the shrinkage rate of MD and V was found between pCR and non-pCR (*P* = 0.001; *P* = 0.001, respectively). Change of the parameters between pCR and non-pCR are summarized in Table 4 and Figure 3.

**TABLE 4:**
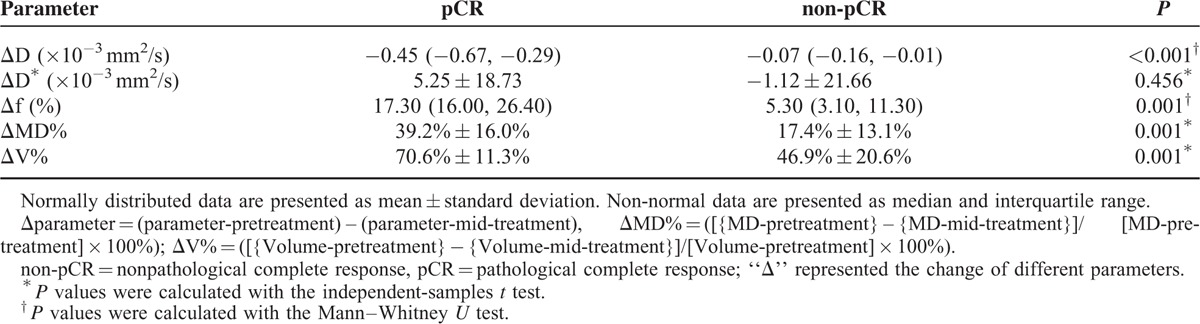
Change of Parameters Between pCR and non-pCR

### Diagnostic Performance for Predicting Response

**FIGURE 4:**
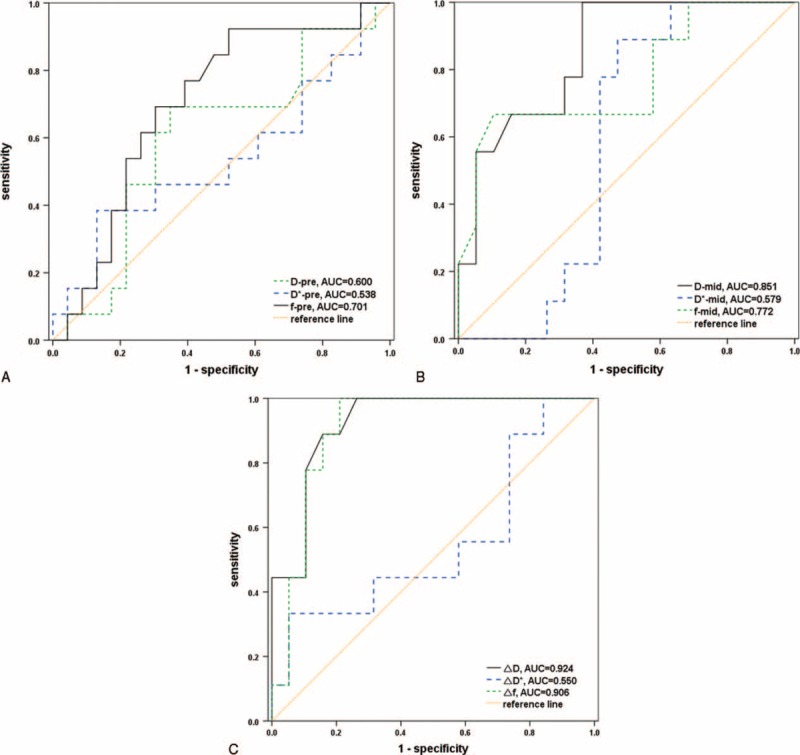
Receiver-operating characteristic curve analyses of parameters deriving from IVIM model and their changes for the prediction of the pathologic responses. The area under the curve (AUC) of the D, D^∗^, and f value in pretreatment (D-pre, D^∗^-pre, f-pre) was 0.600, 0.538, and 0.701, respectively. The AUC of the D, D^∗^, and f value after 2 cycles of NAC (D-mid, D^∗^-mid, f-mid) was 0.851, 0.579, and 0.772, respectively. The AUC of the change of parameters (Δparameter) after the chemotherapy was 0.924 for ΔD, 0.550 for ΔD^∗^, and 0.906 for Δf.

During the mid-treatment time point, the D showed an excellent diagnostic performance of prediction by the area of the curve 0.851 (95% CI = 0.666–0.956), which was slightly higher than the D^∗^ value (AUC = 0.579, 95% CI = 0.379–0.762, *P* = 0.025). Meanwhile the f value showed a reasonable diagnostic performance (AUC = 0.772, 95% CI = 0.575–0.908). The optimal cutoff of D during the NAC to differentiate pCR from non-pCR was 0.971 × 10^–3^ mm^2^/s, which yielded a sensitivity of 100% (95% CI = 66.4%–100%) and a specificity of 63.2% (95% CI = 38.4%–83.7%). ROC analysis showed that the AUCs for distinguishing pCR group from non-pCR group were 0.924 (95% CI = 0.759–0.990) for ΔD value, 0.550 (95% CI = 0.352–0.737) for ΔD^∗^ value, 0.906 (95% CI = 0.735–0.983) for Δf value, 0.848 (95% CI = 0.662–0.955) for the reduction ratio of MD, and 0.865 (95% CI = 0.684–0.964) for the reduction ratio of V. Obviously, the AUC of the increase of D value was the highest, although no statistically significant difference was found among the vast majority of parameters(ΔD vs ΔD^∗^, *P* = 0.004, Δf vs ΔD^∗^, *P* = 0.004, ΔMD% vs ΔD^∗^, *P* = 0.024, ΔV% vs ΔD^∗^, *P* = 0.016; *P* > 0.05 for other groups). If the growth of D value was set at −0.163 × 10^−3^ mm^2^/s, the values for sensitivity and specificity were 100% (95% CI = 66.4%–100%) and 73.7% (95% CI = 48.8%–90.9%), respectively. The results of ROC curve analyses for all parameters are summarized in Table 5 and Figure 4.

**TABLE 5:**
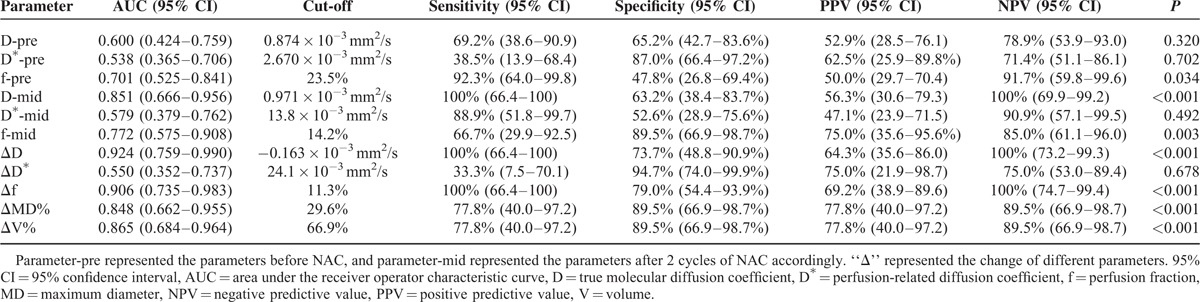
Diagnostic Performance for Predicting Pathologic Response

**FIGURE 5:**
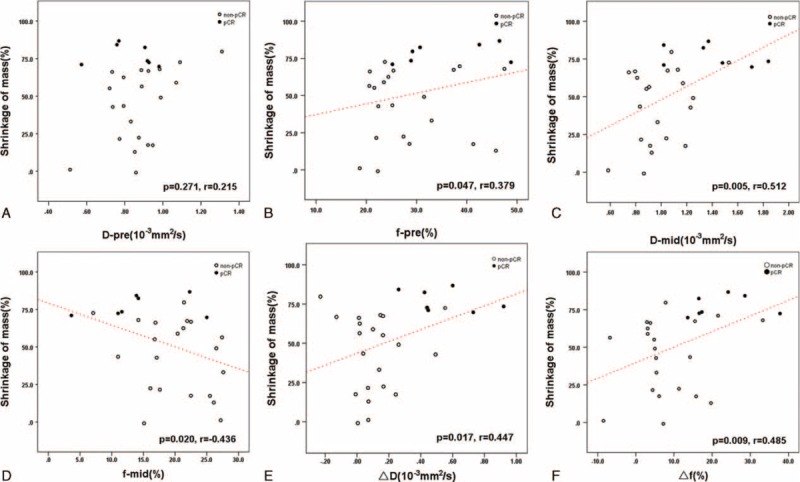
Scatter plots of the mass shrinkage percentage versus D-pre(A), f-pre(B), D-mid(C), f-mid(D), ΔD(E), and Δf(F). The *P* values and corresponding correlation coefficient (*r* value) for the spearman correlation tests were represented for each plot. The linear least-squares fit was depicted if there was a significant correlation.

The correlation between the IVIM-derived parameters with the mass shrinkage can be seen in Figure 5. Before the treatment, no statistically significant correlation was found between the mass shrinkage and the baseline D (*P* = 0.271), whereas a positive correlation was observed between the shrinkage of mass and the f value (*P* = 0.047). In the mid-treatment, positive and negative correlations were observed between the mass shrinkage and D (*P* = 0.005) and f (*P* = 0.02), respectively. Both the change of D (*P* = 0.017) and f (*P* = 0.009) demonstrated positive correlation with the mass shrinkage. Overall, it can be seen that mid-treatment D and change of f showed the best correlation with the effects of NAC.

## DISCUSSION

Pretreatment prediction and early monitoring of the histopathologic response to chemotherapy is desirable for developing an individual and optimal regimen of NAC for regionally advanced breast carcinoma patients. Some researchers suggested that breast cancer with lower pretreatment ADC values have achieved better treatment response,^24,25^ while some studies did not find a significant correlation between pretreatment ADC and pathologic response.^26–28^ Hence, the use of ADC as an early biomarker for pathologic response is still controversial. Multiple b value based IVIM model allows the separation of pure water diffusion and microcirculation perfusion, and may have better potential for NAC effects prediction. In our study, there is no statistical difference observed in D value before the NAC between the pCR and non-pCR group, but the f value in pCR is significantly higher than that in non-pCR. By using DCE-MRI, Chawla et al^29^ claimed that tumors with a good prognosis initially showed a higher plasma volume fraction. In our study, a similar observation was made. The f value reflects the process of angiogenesis in immature blood vessels and partly reflects the microvasculature's permeability; as a result, an elevated level of f may indicate better effects of chemotherapy drugs on tumor cells and consequently a better response. Hence, the perfusion fraction f may have the potential ability to differentiate the pathologic response to NAC before treatment.

After 2 cycles of NAC, an increase of D value was observed in our study. This increase of D value after tumor treatment may reflect the reduction of cellularity due to necrosis and fibrotic change as a treatment effect. These findings are in agreement with previous studies that also reported increased ADC values after chemotherapy.^24,30^ Meanwhile, the f value for all subjects significantly decreased during the treatment. Some scholars reported that fewer tumor microvessels were found in breast cancer patients treated with chemoendocrine therapy than in untreated patients.^31,32^ Pickles et al^33^ and de Bazelaire et al^34^ also discovered that the DCE parameters (including K^trans^, K_ep_, or V_e_) were also reduced to various extents in breast cancer patients after NAC. These results are consistent with the observation in our study. After chemotherapy, cytotoxic drugs lead to tumor cell death resulting in a reduction in cell density and immature endothelial cells; extracellular spaces would be enlarged, allowing water molecules to move with less restriction and weakening the process of perfusion.^20,31^ Guo et al^35^ discovered that the f and D^∗^ values significantly decreased and D significantly increased after the radiofrequency ablation in rabbit VX2 tumors. Xiao et al^20^ demonstrated a significantly higher D and lower D^∗^ value after NAC in nasopharyngeal cancer, although with a more consistent f value. In our study, a significantly higher D and lower f value was seen after NAC, which is partially consistent with the result of Guo et al.^35^ However, in our finding, the decrease of D^∗^ showed no statistically significant difference. This could be attributed to the large level of fluctuation of the D^∗^ measurements in our study, which may overwhelm the relatively small level of decrease of D^∗^. Further study on confirming the reproducibility of D^∗^ value should be performed to confirm the change of D^∗^.

During the early treatment, the D value was significantly higher in pCR than that in non-pCR. Similarly, Fangberget et al^36^ showed that mid-treatment ADC was higher in those patients who had pCR than those without pCR. In addition, in this work, the f value was significantly lower in the pCR patients, although no significant differences are found in the MD and V between the pCR and non-pCR groups. So, the D and f value in the mid-treatment may be 2 independent predictors for the pathological response, when the morphological changes are not significantly different among the 2 groups. This discovery would be of great clinical interest; however, further study with a larger sample size would be needed for more solid evidence.

Reduction rate of tumor size is the most commonly used means for evaluation of treatment response. In this study, an obvious reduction in MD and volume of tumor as measured using DCE-MRI was found after 2 cycles of NAC. There were significant differences between the pathologic responsive and nonresponsive groups, which was consistent with previous reports.^37,38^ In addition, the AUC of the volume reduction rate for distinguishing response from nonresponse is higher than that of the longest diameter reduction (0.865 vs 0.848), though no significance difference was found, and similar observations were reported.^38,39^ In the parameters of IVIM model, this present study indicates that the change of D and f value can effectively differentiate the pCR from non-pCR. Tumor cell and microvessel density may decrease due to cytotoxic and antiangiogenic effects of the chemotherapy. On the one hand, there is excessive amount of micronecrosis in tumor cells with the cytotoxic effect of chemotherapeutic agents. Pure water diffusion motion becomes more unrestricted because of bigger extracellular and extravascular spaces, which increases the D value. Therefore, there is a more significant increase of the D value, owing to a better chemotherapeutic response to NAC. On the other hand, the f value is mainly related to the volume fraction of microcirculation.^12^ Successful chemotherapy can cause cytotoxic tumor cell death resulting in a fall in the proportion of immature microvessel density in breast tumors.^40^ So, microvascular structures would decrease more dramatically, resulting in a more significant reduction of the f value and a better chemotherapeutic response to NAC. At the same time, a trend toward higher change of D^∗^ value could be noticed in the pCR group patients, though there is no significant difference between the 2 groups. These suggest that the change of IVIM-MRI parameters can be a potential predictor of the chemotherapeutic response for breast cancer at the early stage of NAC. The early prediction and monitoring ability of IVIM parameters is also supported by the correlation between the parameter values and the effects of NAC, as judged by the shrinkage of mass. It was observed that, among pretreatment, mid-treatment, and change of D and f measurements, middle treatment D and change of f were most sensitive to the shrinkage of mass, whereas pretreatment D showed no statistically significant correlation with the shrinkage of mass.

By comparing the AUC among the parameters during the NAC, the increase of the D value had the best predictive performance for distinguishing between the pCR and non-pCR in present study. The diagnostic sensitivity and specificity of D value was as high as 100% and 73.7%, respectively, when the optimal cutoff value was −0.163 × 10^−3^ mm^2^/s. However, the diagnostic performance of the shrinkage rate of the MD and V was lower than that of the change of D value, especially the diagnostic sensitivity. During the chemotherapy, malignant tissue cells in many breast carcinomas are reduced and become necrosis in the form of a sieve, which results in later and smaller changes of tumor size than the diffusion parameters. Many previous researches^41,42^ demonstrated that the increase of diffusion parameter was a significant difference between the responsive and nonresponsive patients, whereas not in the decrease of tumor size after 1 cycle of chemotherapy. In our study, meanwhile, the decrease of the f value and the mid-treatment D value also showed reasonably good diagnostic performance (AUC = 0.906; 0.851, respectively). On the basis of the results in present study, we suggest that IVIM-derived parameters (including D and f value) have excellent prediction performance for pathologic response.

There are some limitations in our preliminary study. Firstly, breast cancer is a type of heterogeneous tumor, which has 4 distinct phenotypes; the treatment response closely depends on the breast tumor phenotype. We did not assess the prediction efficiency between different pathologic responses according to molecular subtype on account of our small population. Our study at the next stage will be focused on evaluating the pretreatment prediction and early response monitoring ability of IVIM-derived parameters on different molecular subtypes. Secondly, in this study, the time point for early monitor of the treatment was set in the second cycle of NAC, when most tumors, both responsive and nonresponsive ones, may have morphological changes in the time point. We should adjust our study in future to choose an earlier time point, such as 3 days or 1 cycle after NAC, in order to investigate the prediction value of IVIM model parameters before the change of the lesion size. Finally, the sample size of this study was relatively small for such a prospective analysis that may lead to a statistical bias. So, a larger sample cohort and perhaps multicenter research is preferable for the next phase research.

In conclusion, IVIM-derived parameters, especially the D and f value, played an important role in the pre-treatment prediction and early response monitoring to NAC in locoregionally advanced breast. Patients who had a higher baseline f value, a higher mid-treatment D value, and a lower mid-treatment f value were observed to respond better to NAC. Patients in the pCR group had a larger change of D and f value than those in the non-pCR group. The prediction performance of the change of D value for distinguishing the pCR from non-pCR was excellent after 2 cycles of NAC. D and f value derived from IVIM model showed the potential to be used for early treatment prediction and response monitoring.
